# Neural Representation in mPFC Reveals Hidden Selfish Motivation in White Lies

**DOI:** 10.1523/JNEUROSCI.0088-21.2021

**Published:** 2021-07-07

**Authors:** JuYoung Kim, Hackjin Kim

**Affiliations:** Laboratory of Social and Decision Neuroscience and School of Psychology, Korea University, Seoul, 02841, Republic of Korea

**Keywords:** decision making, dishonesty, fMRI, mPFC, moral decisions, prosociality

## Abstract

Identifying true motivation for Pareto lies, which are mutually beneficial for both the liar and others, can be challenging because different covert motivations can lead to identical overt behavior. In this study, we adopted a brain-fingerprinting approach, combining both univariate and multivariate analyses to estimate individual measures of selfish motivation in Pareto lies by the degree of multivoxel neural representation in the mPFC for Pareto lies conforming with those for selfish versus altruistic lies in human participants of either sex. An increase in selfish motivation for Pareto lies was associated with higher mean-level activity in both ventral and rostral mPFC. The former showed an increased pattern similarity to selfish lies, and the latter showed a decreased pattern similarity to altruistic lies. Higher ventral mPFC pattern similarity predicted faster response time in Pareto lies. Our findings demonstrated that hidden selfish motivation in white lies can be revealed by neural representation in the mPFC.

**SIGNIFICANCE STATEMENT** True motivation for dishonesty serving both self and others cannot be accurately discerned from observed behaviors. Here we showed that fMRI combining both univariate and multivariate analyses can be effectively used to reveal hidden selfish motivation of Pareto lies serving both self and others. The present study suggests that selfish motivation for prosocial dishonesty is encoded primarily by increased activity of the ventromedial and the rostromedial prefrontal cortex, representing intuitive self-serving valuation and strategic switching of motivation depending on beneficiary of dishonesty, respectively.

## Introduction

The consequences of dishonest behavior regarding oneself or others are the key elements that drive dishonesty. Recent studies have reported neural processes associated with prosocial and selfish goals of dishonesty (Yin et al., 2017; Cui et al., 2018). However, less is known about Pareto lies (Erat and Gneezy, 2012), where the results of dishonesty are mutually beneficial for both the liar and others. Two different psychological mechanisms have been proposed to contribute to increasing Pareto lies. The presence of another beneficiary (1) may help justify dishonesty that will benefit oneself or (2) may trigger genuine care and concern about the benefits others receive (Gino et al., 2013). As Pareto lies are both self-serving and altruistic, recognizing the exact mechanisms engaged from the dishonest behavior alone poses a challenge.

We applied the concept of brain fingerprinting technique (Ahuja and Singh, 2012) to neuroimaging data to gain further evidence for inferring an individual's covert motivation of Pareto lies. In this approach, a target without an explicit label can be classified based on the degree to which the brain response to the target resembles the two known categories. More specifically, contexts in which dishonesty may benefit both self and others may appear as a selfish opportunity to some as they may benefit from dishonesty, whereas the same context may be viewed by others as an altruistic opportunity to benefit others.

Several subregions of the mPFC serve a crucial role in moral judgment and generation of dishonest behavior. For example, judgments of the dishonesty of a scenario activate the dorsomedial PFC (Parkinson et al., 2011), spontaneous lying engages the subgenual ACC (Yin et al., 2016), and the ventromedial PFC (vmPFC) is involved in deceiving others (Abe et al., 2007) regardless of whether dishonesty is beneficial to the liar or others (Pornpattananangkul et al., 2018). Subregions of mPFC have been reported to represent self- and other-regarding values differently, where the individual differences in prosociality are expressed as the spatial gradient along the dorsal-to-ventral axis in representing self- and other-regarding values (Sul et al., 2015). Importantly, the rostromedial PFC (rmPFC), which includes the pregenual ACC (Vogt, 2005), is known for computing the values of the outcomes that benefit both self and others (Hutcherson et al., 2015; Sul et al., 2015) and of context-dependent strategic social decisions (Jung et al., 2018; Yoon et al., 2018). It was recently suggested that mPFC subregions are hierarchically organized so that more dorsal regions use additional external sensory information from the environment to regulate more ventral subregions that compute intuitive social values based on internal bodily signals (Kim, 2020). From this perspective, we predicted that distinctive patterns of activity across mPFC subregions would reflect individual differences in motivation for Pareto lies, given the roles of vmPFC and rmPFC in intuitive social valuation and context-dependent strategic social valuation, respectively.

**Figure 1.**
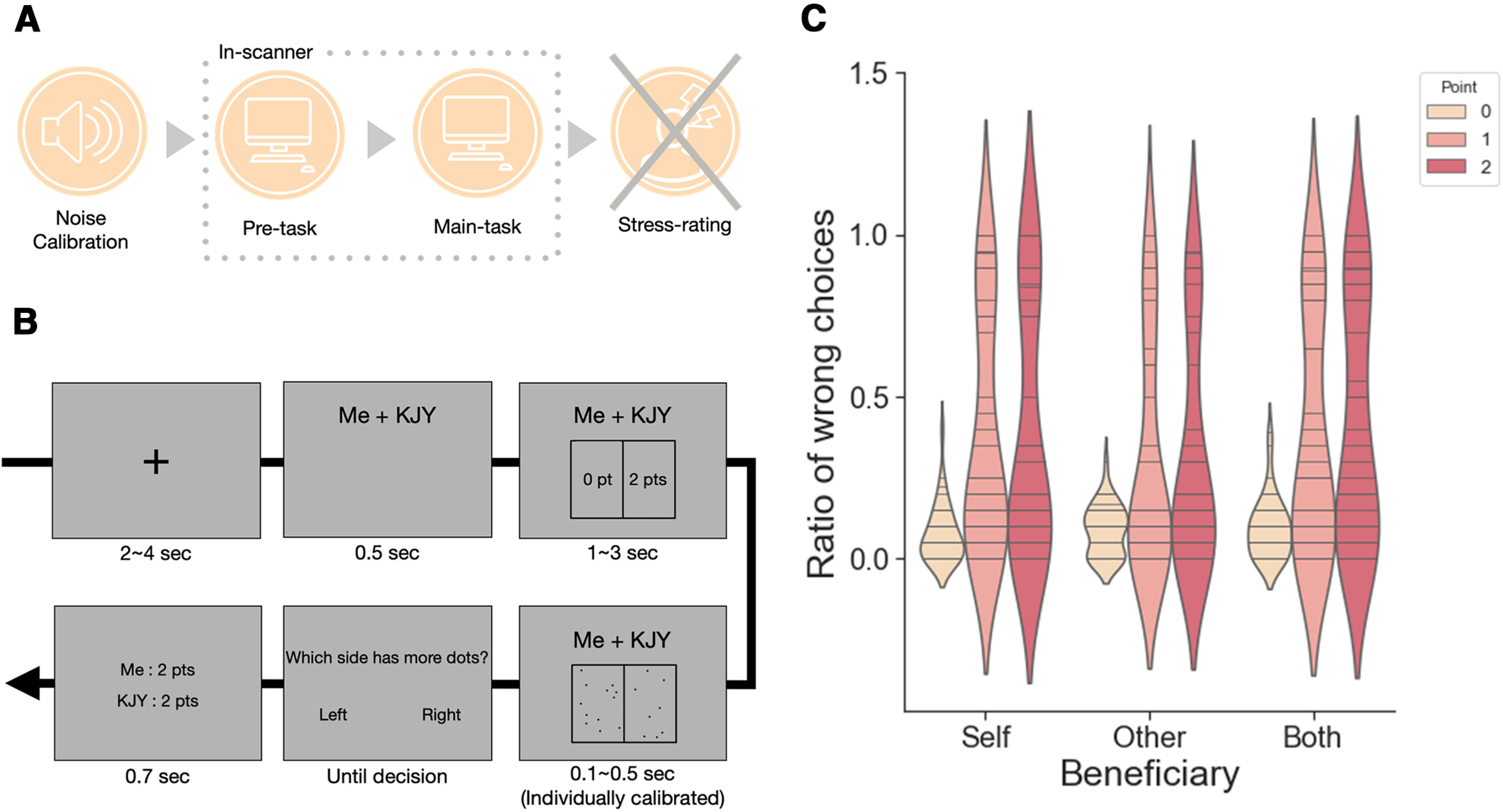
Dot discrimination task. ***A***, The overall flow of the experiment. ***B***, An example of a single trial in the dot-discrimination task. The trial is an example of Both, two-point trial, where dishonesty would earn 2 points for both the participant and the partner. ***C***, Violin plot of the mean probability of choosing the wrong answer for each condition. Each horizontal line represents a participant's mean probability for the respective condition.

We aim (1) to identify individuals' primary motivation behind Pareto lies and (2) to examine neural mechanisms that underlie the processing of immoral opportunities to gain from Pareto lies as opportunities to justify selfish gain, particularly focusing on the differential engagement of the mPFC subregions. To this end, we devised a behavioral task that could measure selfish and altruistic lies as well as Pareto lies. Participants took part in a dot-discrimination task inside the MRI scanner, where they could gain points that would later reduce the length of the stressful task for themselves, another person, or both by being dishonest in each trial (see Fig. 1*B*). We applied both the univariate and multivariate analyses to probe the neural mechanism that underlies the individual difference in the selfish motivation for Pareto lies, as univariate tests may detect the regions mapping the subject-level variability in the selfish motivation but may not be sensitive enough to reveal the latent subfeatures between the conditions within an individual (Davis et al., 2014).

## Materials and Methods

### 

#### Participants

Forty-three participants (16 females, mean age = 23.79 ± 2.49 years) were recruited through Korea University students' community website. The following 7 participants were excluded from the analyses: 3 participants for later reporting not to have believed in the experiment cover story, 2 for misunderstanding the instruction, 1 for reporting a neuropsychological drug's intake, and 1 for sleeping during the main task. Behavioral and neuroimaging data of 36 participants were included in the analyses. A power analysis for a repeated-measures ANOVA testing for within factors suggested that the appropriate sample size to achieve a power of 0.95 with an α of 0.05 and an effect size of 0.31 was 32. The effect size used in the power analysis was calculated from the partial η^2^ of beneficiary × point interaction taken from an independent behavioral pilot study. All participants gave written consent before participation and were compensated with KRW 30,000 (roughly equivalent to USD 30). The study design and the data collection procedures complied with all relevant ethical regulations and were approved by the Korea University Institutional Review Board.

#### Experimental design and statistical analyses

##### Experimental procedure

Participants were given the following overall instruction and a cover story on arrival. We informed all the participants that the study was about the change in the subjective experience of stressful noise after the depletion of cognitive resources resulting from performing a cognitive task that requires attention. They were to be exposed to an aversive noise for 10 min and would have to report the stressfulness of the noise after the main task in the MRI scanner. The subject-specific noise level participants would be later exposed to were determined through the noise thresholding procedure to ensure that every subject would experience the same level of evoked stress. They would earn the points in the main cognitive task for themselves or their partner, and the earned points were to be used to reduce the duration of exposure to the stressful noise for the respective beneficiary. The partner they were obtaining points for was another person, unknown to the participant, that would participate in the same experiment immediately after the participant. All participants were told that the same procedure was done for the previous participant, but the amount of points obtained by the previous participant for them was untold. Reduction of the stressful task was used instead of monetary gain as reward for dishonest gain, because controlling for the subjective value of each point across participants in the absence of beneficiary was crucial as our goal was to observe differences in the motivation behind dishonesty for different beneficiaries. The value of each point was manipulated to be similar across participants through the noise thresholding procedure.

Following the overall instruction (see Fig. 1*A*), each participant went through the noise thresholding and the dot-screen display time calibration procedures before participating in the main task. Dot-screen display time calibration and the main task were performed inside the scanner. We introduced the dot-screen display time calibration procedure as practice trials. The stressfulness rating, which participants believed they would have to participate in after the main task, did not actually take place.

##### Noise thresholding procedure

Participants listened and evaluated a series of brief sounds with differing frequency and volume on a 10 point averseness scale. Participant-specific noise thresholds were determined as the sound each participant evaluated as 8 on the 10 point averseness scale. This procedure allowed controlling for the subjective value of points to be obtained during the main task.

##### Dot-screen display time calibration procedure

Participants performed a simpler version of the dot-discrimination task before the main task. They were asked to report the side with more dots. We lengthened the dot screen display time duration when the participant reported the wrong side on the previous trial until each participant could provide correct answers in 10 consecutive trials. The final length of the display time determined by this procedure was used as the dot-screen display time customized for each participant in the main task. We adopted this procedure to ensure that the participants' dishonest decisions in the main task were the intended dishonesty, rather than a perceptual mistake. However, this procedure was introduced as a practice trial, and participants were unaware of the intention behind the procedure.

##### Dot-discrimination task

The task was introduced to the participants as a visual perception and attention task, and participants were instructed to report the side of the screen with more dots. In each trial, the beneficiary and the points assigned to each side of the screen were shown before the dot-screen appeared. The reward magnitude (i.e., number of points) and the beneficiary of the dishonest decision were experimentally manipulated and varied across trials. We displayed the dot-screen for the individually calibrated length of time, which was just long enough for the participant to be aware of the difference in the number of dots between the two sides. Points could only be obtained by being dishonest, that is, by choosing the side with fewer dots, and could benefit the participant (Self), their partner (Other), or both the participant and the partner (Both) (see Fig. 1*B*). The number of points ranged from 0 to 2 points, and the points obtained in the five randomly selected trials across conditions were to be used to reduce the duration of the exposure to the stressful noise after the task; and each point would reduce 10 s of the total duration. Twenty trials existed per each condition, resulting in 180 trials in total. Thus, a single trial consisted of a fixation period (2-4 s), the beneficiary information display (0.5 s), followed by the number of points assigned to each side of the screen (1-3 s), dot-screen display for the individually calibrated length of time, question display (until decision), and the result of choice display (0.7 s).

#### Behavioral data analyses

The overall effect of point and beneficiary on dishonest decisions was assessed by entering the percentage of wrong choices to a repeated-measures ANOVA with the beneficiary (Self, Other, Both) and point (0, 1, 2) as within-subject factors. As the points could only be obtained by reporting the wrong answer, we expected a higher percentage of dishonest decisions in Points 1 and 2 conditions compared with point 0 condition.

We first normalized the response time (RT) data within each subject over all trials, and then averaged them separately for dishonest decisions in each condition. For participants who were always honest in certain conditions and whose average RT could not be calculated were excluded from correlation analyses that includes RT data. The correlation between RT data and other indices was obtained using Spearman's rank correlation as the sample size after exclusion resulted in 28, which may be insufficient to use Pearson's correlation. The average normalized RT of each condition was calculated and entered in the repeated-measures ANOVA for all 36 participants.

#### Neuroimaging procedures and analyses

##### fMRI data acquisition and preprocessing

fMRI data were acquired using a 3.0 T Siemens Magnetom Trio MRI scanner with a 12-channel head matrix coil located at the Korea University Brain Imaging Center. We obtained the T2*-weighted functional images using gradient-echo echo-planar pulse sequences (TR = 2000 ms; TE = 30 ms; flip angle = 90, FOV = 240 mm, 80 × 80 matrix; 36 slices; voxel size = 3 mm × 3 mm × 3 mm). The fMRI BOLD activity was measured over one functional run, lasting ∼25 min. We acquired the EPI volumes at an oblique angle to the AC-PC line to decrease the impact of susceptibility artifacts in the orbitofrontal cortex. High-resolution T1-weighted (TR = 1900 ms; TE = 2.52 ms; flip angle = 9; 256 × 256 matrix; 1 × 1 × 1 mm in-plane resolution) structural images and diffusion tensor scans (TR = 3000 ms; TE = 70.0 ms; 224 × 224 matrix; voxel size = 2 mm × 2 mm × 2 mm) were also obtained. The stimuli were presented through an MR-compatible liquid-crystal display monitor mounted on a head coil (refresh rate: 85 Hz; display resolution: 800 × 600 pixels; viewing angle: 30 horizontal, 23 vertical).

We preprocessed the data using the SPM12 (Wellcome Department of Imaging Neuroscience, University College London). Images were temporally corrected for interleaved slice acquisition and then realigned to the first volume to correct for head motion, and a mean image was created for each participant. The realigned images were normalized to the standard MNI EPI template, resampled to 2 × 2 × 2 mm voxels, and spatially smoothed using a Gaussian kernel with an 8 mm FWHM.

##### First-level univariate analyses

A first-level GLM was estimated to create contrasts for each beneficiary condition. Onset times for the three beneficiaries (Self, Other, and Both), with the three points (0, 1, and 2 points) information presentation and decisions for each nine condition as well as six head-motion parameters were included as regressors after being convolved with a standard HRF. The brain regions reflecting the point × beneficiary interaction effect were identified by first generating three contrast images (i.e., one for each beneficiary condition) by combining Point 1 and 2 conditions and subtracting Point 0 condition at decision onset (e.g., [Point 1 + Point 2] − Point 0 for Self condition), and then entering the contrasts into a repeated-measures ANOVA. These three contrasts were used in the pattern classification analyses as well. We used these contrasts rather than the contrast of dishonest versus honest decisions because (1) some participants do not have enough trials of dishonest decision in some conditions, and (2) the focus of this research was to distinguish individual motivation and neural mechanisms that underlie the processing of immoral opportunities to gain from Pareto lies.

##### Second-level univariate analyses

To explore brain regions representing the main effects of beneficiary and point, and the interaction effect between beneficiary and point, three repeated-measures ANOVAs were conducted. The beneficiary main effect was assessed by constructing first-level contrast images for each beneficiary at decision onset by combining trials overall points for each beneficiary (i.e., Point 0 + Point 1 + Point 2 separately for each of Self, Other, and Both), which were entered into a repeated-measures ANOVA. In addition, contrasts for each point overall beneficiaries were built and entered into a repeated-measures ANOVA to examine the main effect of the points. All the statistical maps reported were thresholded at the whole-brain FWE-corrected *p* < 0.05 at voxel level.

##### Neural signatures of selfish or altruistic motivation for dishonesty: multivariate analysis

A total variation L1 (TV-L1) pattern classifier (Gramfort et al., 2013) was trained to distinguish between neural patterns associated with the opportunities to lie for Self and Other at the moment of decision. The analysis was performed with Nilearn and nltools library in Python 3 (Abraham et al., 2014). For each beneficiary, representations in the mPFC of the dishonest opportunities were obtained from individual contrasts combining Point 1 and 2 conditions and subtracting Point 0 condition at decision onset (e.g., [Point 1 + Point 2] − Point 0 for Self condition). As the primary aim of this analysis was to identify individuals' motivation when dishonest opportunities were given to gain for Both, we contrasted the conditions in which participants were motivated to lie (i.e., points would be given when lying) with the condition in which participants had no reason to lie (i.e., no point would be given when lying) for each beneficiary. Supporting our rationale for this analysis, the behavioral data showed that participants were induced to lie by the existence, rather than the amount, of available point to be earned. The classifier was first trained on the mPFC activity pattern for Self and Other beneficiary conditions to distinguish between neural patterns associated with the opportunities to lie for Self and Other. Conducting classification using moderately smoothed data is thought to be effective (Op de Beeck, 2010; Hendriks et al., 2017), especially when the objective of the classification is to generalize across subjects (Chang et al., 2015; Weaverdyck et al., 2020). The mPFC binary mask was taken from a meta-analysis segregating mPFC into subregions based on the each region's functional coactivation maps (De La Vega et al., 2016). Of the nine mPFC subregions, we excluded the supplementary motor area and pre-supplementary motor area from the mask as activity related to movements or movement control was not considered in this study. Eightfold nested cross-validation was applied where 10 contrast images of 72 were held out as test data, and the remaining 62 images were used as the training data at each fold. The best performing weights were selected at each fold, and the final classifier weight map was constructed by taking the average of the weights of the overall folds. The mPFC activity pattern of the individual first-level contrast maps of Both condition was entered into the trained classifier to predict the class of each individual's mPFC activity pattern of Both condition (see Fig. 3*A*).

The individual measure of selfish motivation in Pareto lies was defined as how certain each individual's mPFC activity pattern during the Both condition was classified as Self. As such, the signed distance of individual Both contrast to the hyperplane separating Self and Other was calculated and used as the self-class confidence scores (SCCSs), where a higher score translates into higher certainty of being classified into Self. Computationally, this score was calculated by taking the dot product of individual Both contrast and the classifier weight map and adding the intercept term.

##### Second-level regression and correlation analyses with the SCCS

Multiple regression analyses were performed to explore the neural mechanisms behind selfish motivation in each beneficiary's opportunities. In these analyses, the SCCSs were regressed on the contrast maps of Self, Other, and Both conditions separately.

##### Representational similarity analyses

The vmPFC, rmPFC, and precuneus masks were generated from the result of the whole-brain FWE-corrected multiple regression analysis of the SCCS with Both contrasts (vmPFC cluster peak: *x* = −2, *y* = 46, *z* = −8; rmPFC cluster peak: *x* = 8, *y* = 34, *z* = 14; precuneus cluster peak: *x* = 10, *y* = −60, *z* = 34). We extracted the neural activity of Self and Both conditions in the ROIs for each participant from the Self, Other, and Both contrasts used in the univariate analyses. We also calculated the pattern similarity as the Kendall's tau (Popal et al., 2019) between the neural activity patterns in each ROI of Self and Both conditions, and those of Other and Both conditions for each participant. Then, the calculated pattern similarities were correlated with the SCCS.

## Results

### Behavioral results

We first tested whether participants were more likely to report incorrectly when points were available, as this suggests dishonesty, and whether such dishonesty is modulated by the beneficiary of the points. We conducted a two-way repeated-measures ANOVA to assess the effect of points and beneficiary on the participants' decisions to be dishonest. A significant main effect of point (*F*_(2,70)_ = 26.971, *p* < 0.001) was revealed with a significant linear trend (*F*_(1,35)_ = 28.380, *p* < 0.001; Fig. 1*C*) as expected. This suggests that participants were more dishonest as more points were available. The main effect of the beneficiary was also significant (*F*_(2,70)_ = 5.078, *p* = 0.009), and behavioral patterns indicated that the participants were generally more dishonest when points were available for Self or Both than for Other. Beneficiary × point interaction was significant (*F*_(4,140)_ = 3.075, *p* = 0.018), implying that each point had a different impact on dishonest decisions depending on the beneficiary. For each pair of the beneficiaries, we ran a 2 × 3 repeated-measures ANOVA with beneficiary and point as factors to investigate the cause of the interaction. The analyses revealed that the beneficiary × point interaction was significant for Self and Other (*F*_(2,70)_ = 4.894, *p* = 0.010), and Other and Both conditions (*F*_(2,70)_ = 3.722, *p* = 0.029), but not for Self and Both conditions (*F*_(2,70)_ = 0.278, *p* = 0.758). We tested for the difference of two-way interaction terms between pairs of conditions. The interaction of beneficiary and point was calculated as (P1 + P2)/2 − P0 for each beneficiary, and the difference of the interaction term between each pair of beneficiaries was entered in paired *t* tests. The analyses revealed that two-way interaction of points and beneficiary of Self and Other condition pair was significantly different from the two-way interaction of Self and Both condition pair (*t*_(36)_ = −2.514, *p* = 0.017), and the two-way interaction of Other and Both condition pair was significantly different from Self and Both condition pair (*t*_(36)_ = 2.854, *p* = 0.007). This indicates a selectively lower dishonesty rate for Other as opposed to Self and Both conditions. The main effect of the point and beneficiary, and the interaction of the two were not significant for the RT data, but we observed a significant negative correlation between the ratio of dishonest decisions in the Both condition and the RT of dishonest choices in the Both condition (see Fig. 6*C*; Spearman's ρ_(28)_ = –0.561, *p* = 0.002, two-sided). This suggests that the individuals who were faster in dishonest decisions for Both were more prone to be dishonest in the Both condition.

### Neuroimaging results

#### Univariate analysis result

**Figure 2.**
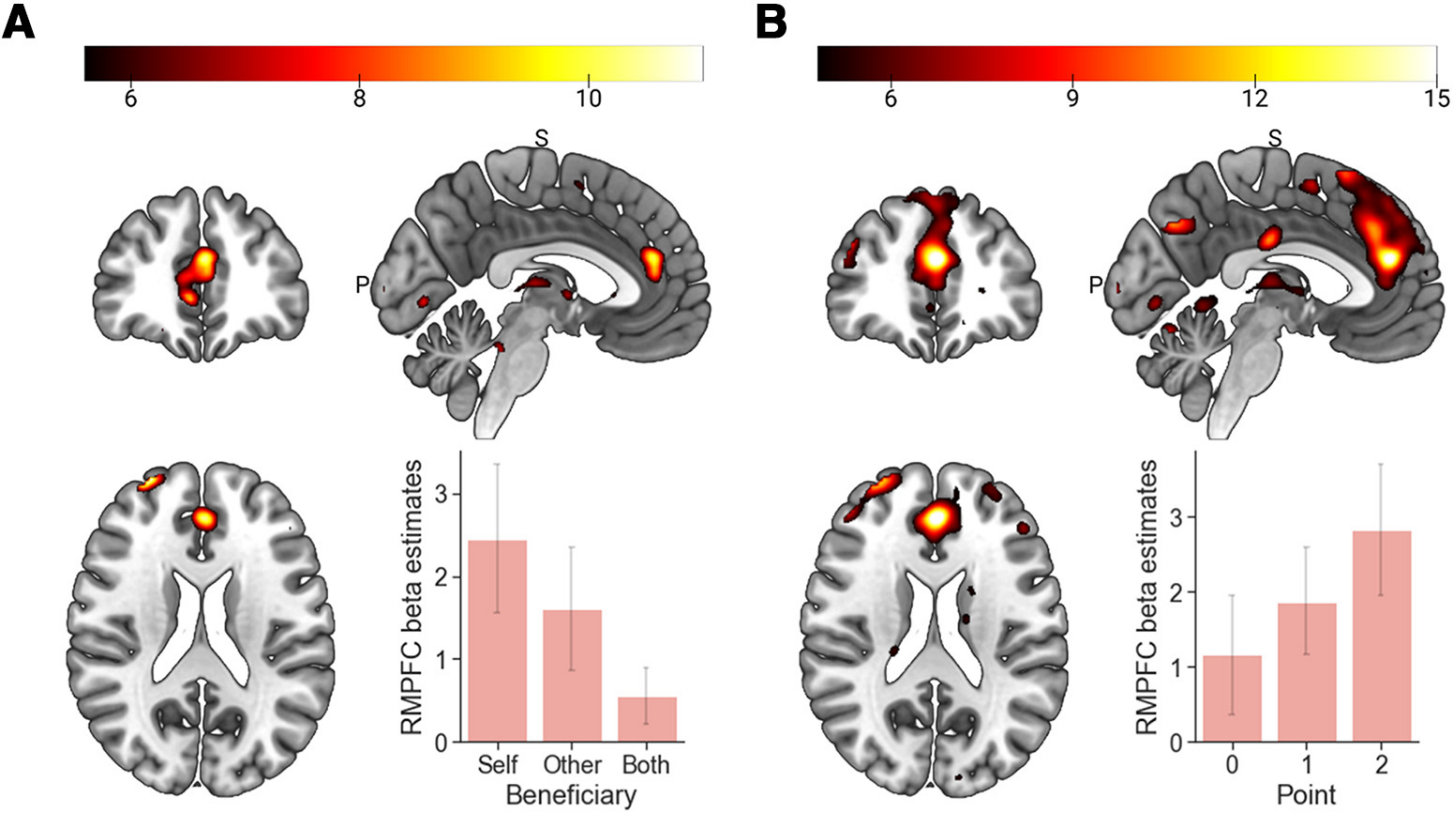
Univariate analysis results. Regions responding differently to different beneficiaries, and amount of points. ***A***, The rmPFC activation was highest when points were available for Self, and lowest when points were available for Both. The results are displayed at the threshold of *p* < 0.05, FDR-corrected at whole-brain level for display purpose. ***B***, The activity in the rmPFC was higher as more points were available. Error bars represent 95% confidence intervals (CIs).

We first investigated how opportunities to gain from dishonesty for different beneficiaries and different amounts of points are represented in the brain. A first-level GLM was built, including onset times for three beneficiaries (Self, Other, and Both), three points (0, 1, and 2 points) information presentation, and decisions for every nine combinations of beneficiaries and points, which were all convolved with a standard HRF. The model also included six motion parameters as nuisance regressors. We created the first-level contrasts for each beneficiary (e.g., Point 0 + Point 1 + Point 2 for Self trials), and each point (e.g., Self + Other + Both for Point 0 trials) to examine brain regions showing the difference in the activation at the time of decision based on the beneficiaries and points, and then entered them into two separate second-level repeated-measures ANOVAs to assess the main effects of beneficiary and points. The analyses revealed a unique rmPFC response for each beneficiary (Fig. 2*A*; *x* = 0, *y* = 40, *z* = 20; whole-brain FWE corrected at voxel-level *p* < 0.05 unless stated otherwise), showing the highest activity during Self, and lowest activity during the Both conditions. Furthermore, a larger rmPFC cluster extending into the dorsomedial PFC was revealed to show differences in the activity to the different amounts of points (Fig. 2*B*; *x* = 0, *y* = 40, *z* = 20), showing higher activity as the available points increased. We assessed the interaction effect between the point and beneficiary using the contrasts constructed by combining conditions where points were available and subtracting the condition where no point was available (i.e., [Point 1 and 2] – Point 0) for each beneficiary for each participant and entering the contrasts into a one-way repeated-measures ANOVA. The interaction between point and beneficiary was also revealed in a large cluster in the posterodorsal mPFC (*x* = 0, *y* = 28, *z* = 42).

#### Neural signatures of selfish or altruistic motivation for dishonesty: univariate analysis

We first conducted a second-level *t* test on Self versus Other contrast and Other versus Self contrast to identify the distinctive neural features related to selfish or altruistic motivation for dishonesty. No voxels survived the correction in both contrasts, which confirms our prediction that a univariate analysis may not be sensitive enough for detecting subtle differences in neural representation between selfish and altruistic motivation for lying.

#### Neural signatures of selfish or altruistic motivation for dishonesty: multivariate analysis

**Figure 3.**
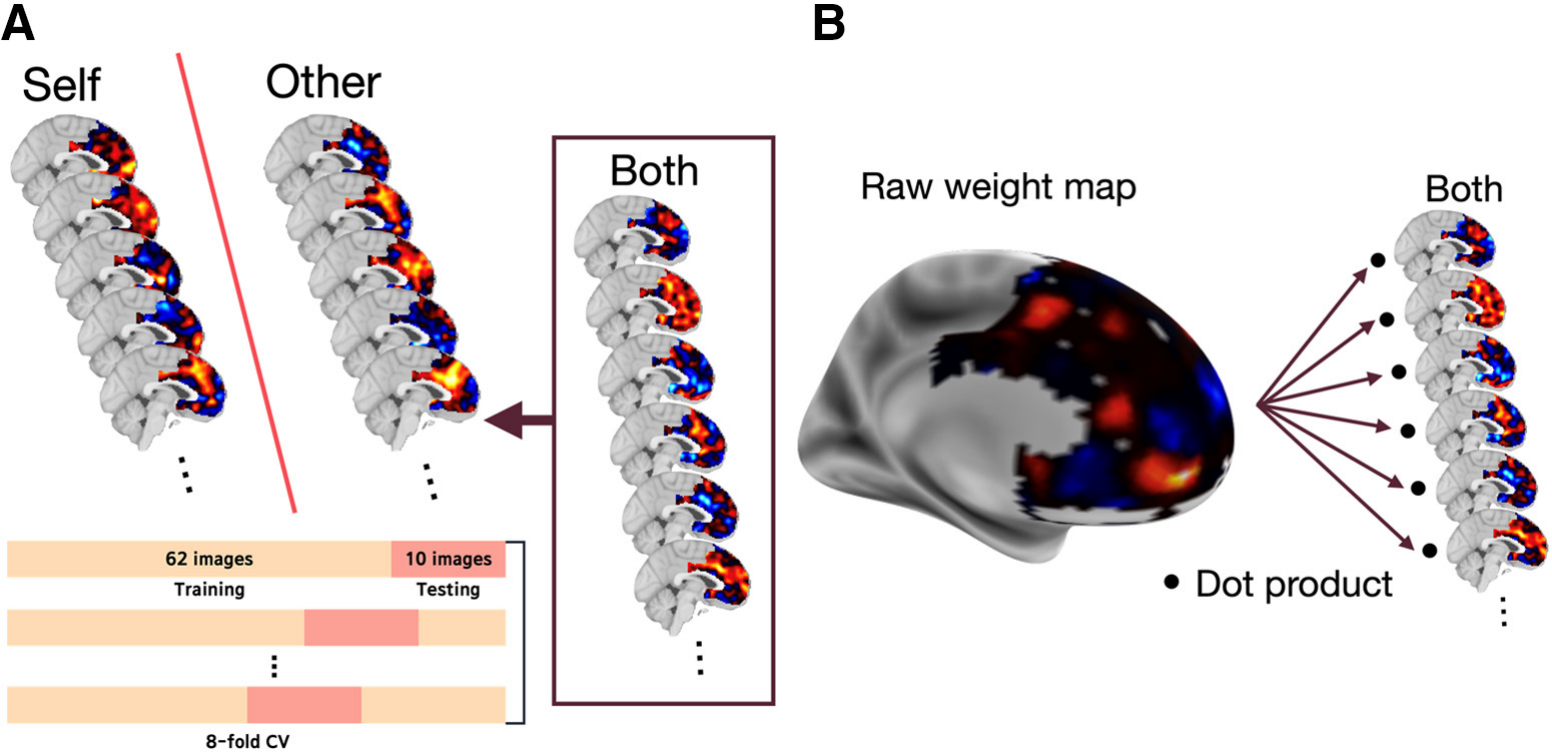
Brain fingerprinting approach ***A***, A pattern classifier was trained to distinguish Self and Other conditions. The trained classifier was then used to forcefully classify Both condition into either of the two classes. ***B***, Each participant's SCCS was calculated by taking the dot product of the classifier weight map and the participant's Both contrast map.

For a further differentiation of the neural signatures of selfish or altruistic motivation for dishonesty in the Both condition, we trained a pattern classifier (for more detailed information, see Neural signatures of selfish or altruistic motivation for dishonesty: multivariate analysis) to differentiate neural patterns in the mPFC associated with the opportunities to lie for Self and Other. We used the trained classifier to classify individuals' neural patterns for Both conditions to estimate one's covert motivation underlying moral decisions in situations where dishonesty would benefit both Self and Other (Fig. 3*A*). The classifier was trained across, rather than within, participants to ensure its generalizability. The final classifier showed 98.61% accuracy in distinguishing Self versus Other contrast images. The classification results showed that Both was classified as Self in 17 of 36 participants and as Other in the remaining 19 participants. The percentage of Pareto lies would not differ between the two groups (*t*_(34)_ = 0.664, *p* = 0.511), consistent with our hypothesis.

#### Neural evidence for selfish motivation in Pareto lies

**Figure 4.**
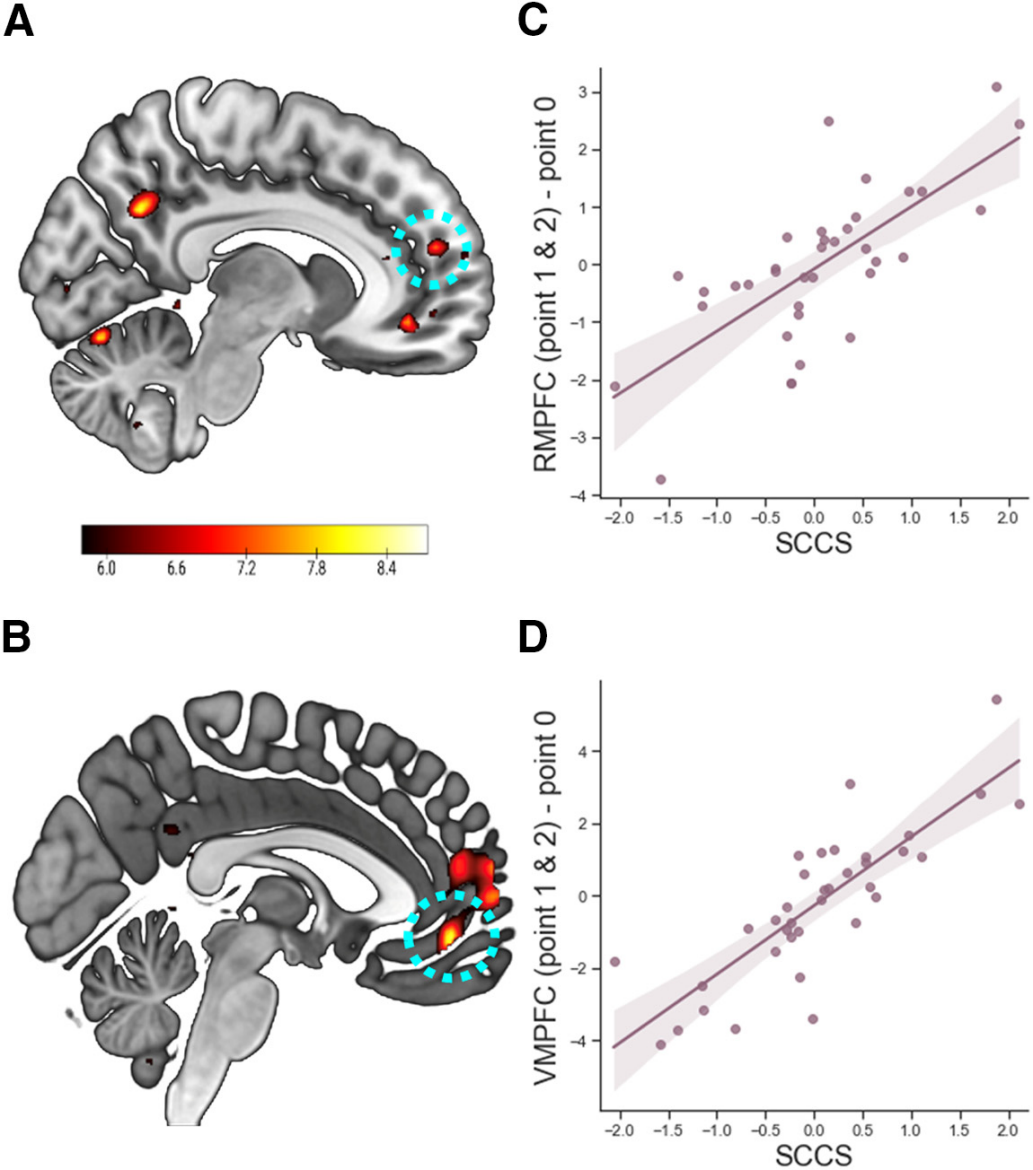
Correlation with SCCS for Both condition. A whole-brain regression analysis where the contrast maps of [Point 1 and 2] versus Point 0 were regressed against the individuals' SCCS shows the clusters with positive correlations in the rmPFC (***A***,***C***) and vmPFC (***B***,***D***). Shaded areas represent 95% confidence intervals (CIs).

Next, we identified neural regions related to the degree of selfish motivation in Pareto lies, which was defined as the SCCS. We calculated the SCCS by taking the signed distance of individuals' Both contrast to the hyperplane separating Self and Other. The SCCS ranged from −2.06 to 2.11 with the mean value of 0.03 and SD of 0.90. The absolute value of the score indicates the certainty of the sample being classified into Self (positive sign) or into Other (negative sign). Thus, the SCCS is assumed to indicate individual differences in the degree of selfish motivation when encountering opportunities to gain from dishonesty for both Self and Other. Individuals' SCCSs were then regressed on the contrast map of the Both condition to identify the neural regions uniquely associated with the opportunity for Pareto lies as a function of the degree of selfish motivation. This analysis revealed that the activities in rmPFC (*x* = 8, *y* = 34, *z* = 14; Fig. 4*A*,*C*), vmPFC (*x* = −2, *y* = 46, *z* = −8; Fig. 4*B*,*D*), and precuneus (*x* = 10, *y* = −60, *z* = 34) positively correlated with the SCCS.

**Figure 5.**
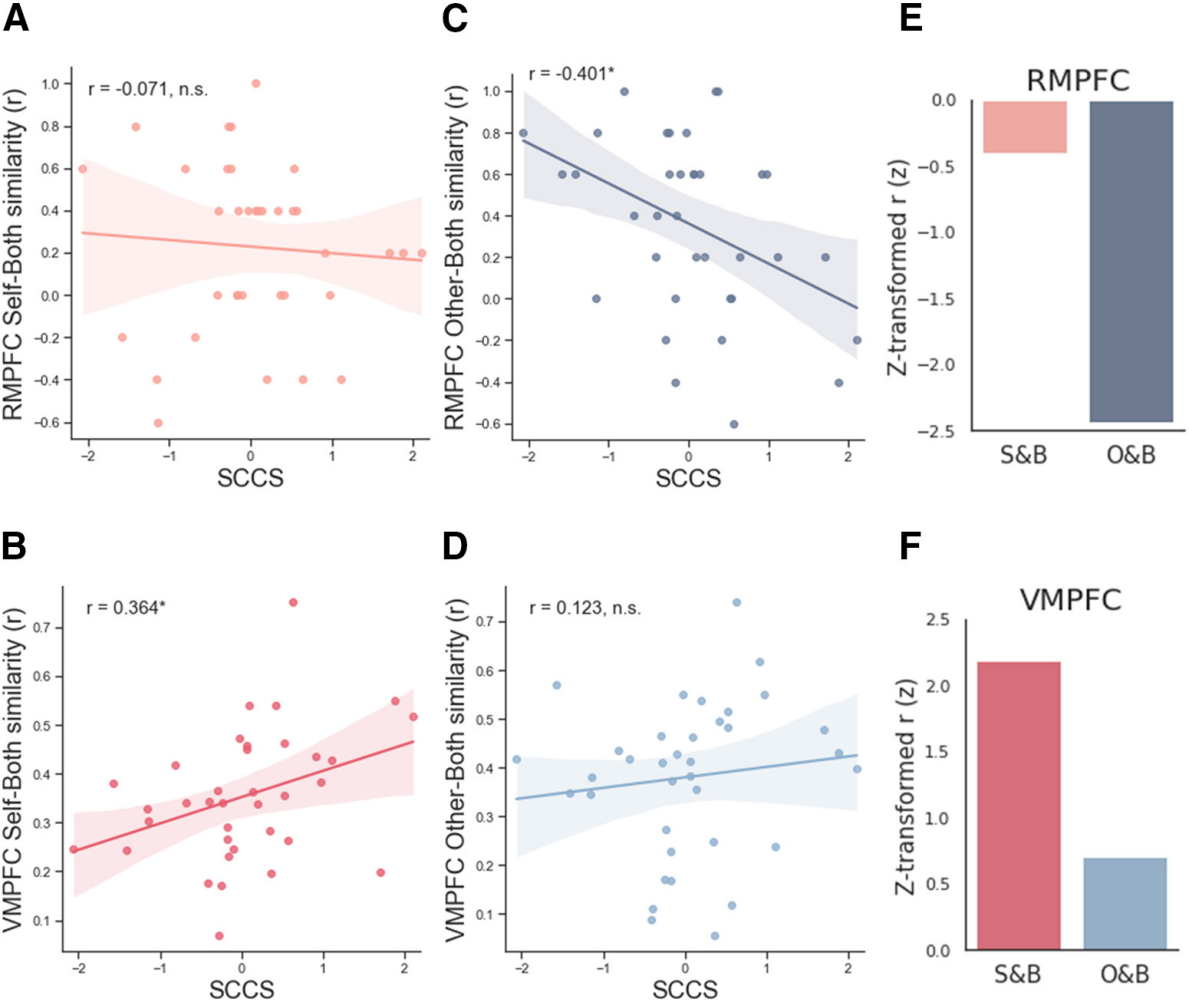
Correlation between the SCCS and the representational similarity between pairs of conditions. In the rmPFC, the SCCS correlated negatively with the degree of pattern similarity between Other and Both conditions (***C***), but not between Self and Both conditions (***A***). In the vmPFC, the SCCS correlated positively with the degree of pattern similarity between Self and Both conditions cluster (***B***), but not between Other and Both conditions (***D***). Fisher's *r*-to-*z* transformed correlation coefficients in the rmPFC (***E***) and vmPFC (***F***). *p < 0.05, n.s. not significant. y-axis values are Kendall's tau values. Shaded areas represent 95% confidence intervals (CIs). r values represent Pearson's r.

Unlike standard univariate tests, multivariate pattern analysis is now known to be insensitive to intersubject variability in mean activation across voxels within an ROI (Davis et al., 2014). Accordingly, we used a representational similarity analysis (RSA) to examine whether multivoxel patterns in each of the three neural regions associated with the SCCS uniquely encode neural evidence for selfish motivation in Pareto lies. As expected, our analysis revealed that the SCCS correlates positively with the degree of similarity of the vmPFC activity pattern between Self and Both conditions (Pearson's *r*_(36)_ = 0.364, *p* = 0.029, two-sided; Fig. 5*B*), but not between Other and Both conditions (Pearson's *r*_(36)_ = 0.123, *p* = 0.475, two-sided; Fig. 5*D*). In addition, the SCCS correlates negatively with the degree of similarity of the rmPFC activity pattern between Other and Both conditions (Pearson's *r*_(36)_ = –0.401, *p* = 0.013, two-sided; Fig. 5*C*), but not between Self and Both conditions (Pearson's *r*_(36)_ = –0.071, *p* = 0.681, two-sided; Fig. 5*A*). Tests for differences in dependent correlations showed that the correlation coefficients of Self-Both similarity and Other-Both similarity in rmPFC cluster are significantly different (*z* = 1.651, *p* = 0.049, one-tailed; Fig. 5*E*), and the correlation coefficients of Self-Both similarity and Other-Both similarity in vmPFC cluster are marginally different (*z* = 1.567, *p* = 0.057, one-tailed; Fig. 5*F*). In the precuneus cluster, the SCCS showed no correlation with the degree of pattern similarity between Self and Both (Pearson's *r*_(36)_ = 0.293, *p* = 0.082) nor between Other and Both (Pearson's *r*_(36)_ = 0.306, *p* = 0.069).

#### Behavioral evidence for selfish motivation in Pareto lies

**Figure 6.**
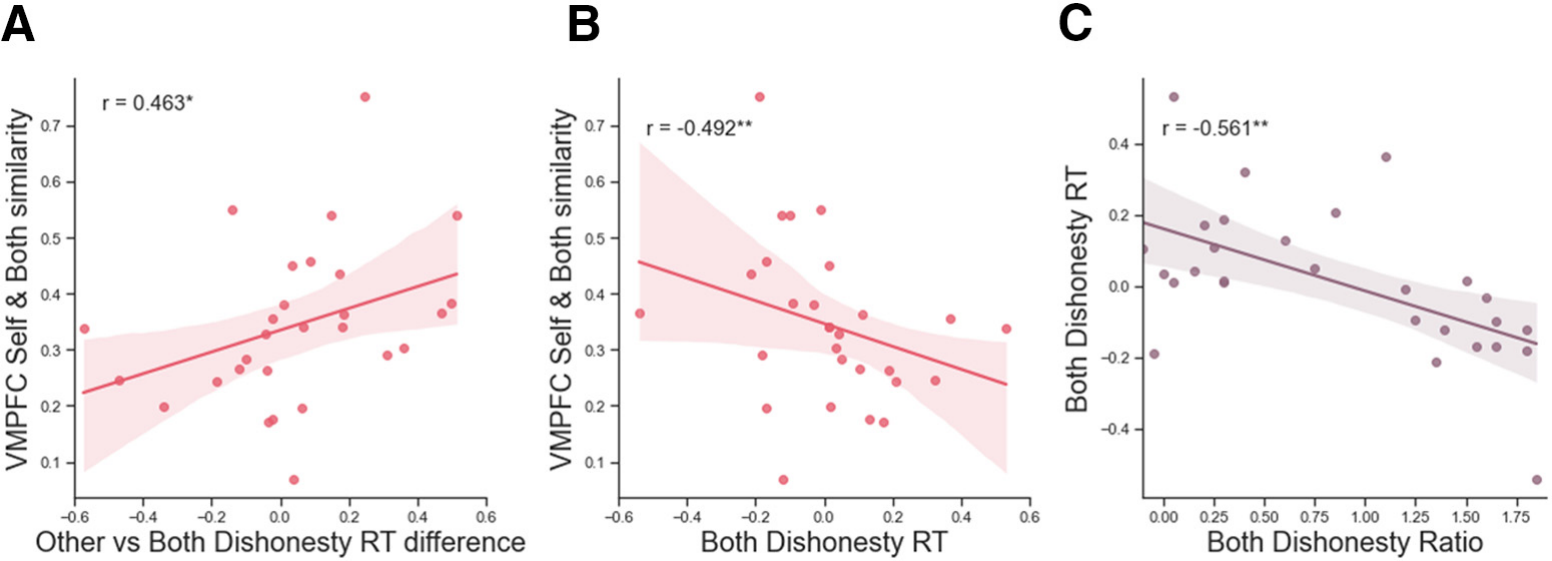
Correlations with RT. ***A***, The RT difference of dishonest decisions for Other versus Both was positively correlated with the neural similarity in the vmPFC between Self and Both conditions. ***B***, The RT of dishonest decisions in the Both condition correlated with the neural similarity in the vmPFC between Self and Both conditions. ***C***, The RT of dishonest decisions in the Both condition correlated with the ratio of dishonesty in the Both condition. *y*-axis values are Kendall's tau values. Shaded areas represent 95% confidence intervals(CIs). r values represent Spearman's rho.

We examined whether this neural evidence for selfish motivation in Pareto lies can be validated by behavioral evidence for Pareto lies. Specifically, we examined the difference between altruistic and Pareto lies as measured by the difference in the proportion of dishonesty and RT between Other and Both conditions. First, as for the vmPFC cluster, the degree of similarity between Self and Both conditions in the activity pattern does not correlate either with the proportion of dishonest choices in the Both condition (Pearson's *r*_(36)_ = –0.066, *p* = 0.699) or with the difference in the proportion of dishonest choices between Other versus Both condition (Pearson's *r*_(36)_ = 0.106, *p* = 0.373, two-sided). However, the same indices show a significant negative correlation with the RT of being dishonest in the Both condition (Spearman's ρ_(28)_ *=* –0.492, *p* = 0.006; Fig. 6*B*) and also a significant positive correlation with RT differences between Other versus Both condition when being dishonest (Spearman's ρ_(28)_ = 0.463, *p* = 0.013, two-sided; Fig. 6*A*). These findings suggest that those with a high degree of selfish motivation in lying for Both engage qualitatively different processes subserving altruistic and Pareto lies, which appears mainly because of their faster intuitive responses in Pareto lies. As for the rmPFC and the precuneus clusters, no significant correlation was found between the representational similarity indices and either Other-Both differences in dishonest decisions (rmPFC: Pearson's *r*_(36)_ = 0.136, *p* = 0.426; precuneus: Pearson's *r*_(36)_ = –0.009, *p* = 0.957) or those in RT (rmPFC: Spearman's ρ_(28)_ = –0.016, *p* = 0.934; precuneus: Spearman's ρ_(28)_ = 0.064, *p* = 0.754).

#### Comparing between selfish and altruistic motivations for dishonesty associated with selfish motivation in Pareto lies

**Figure 7.**
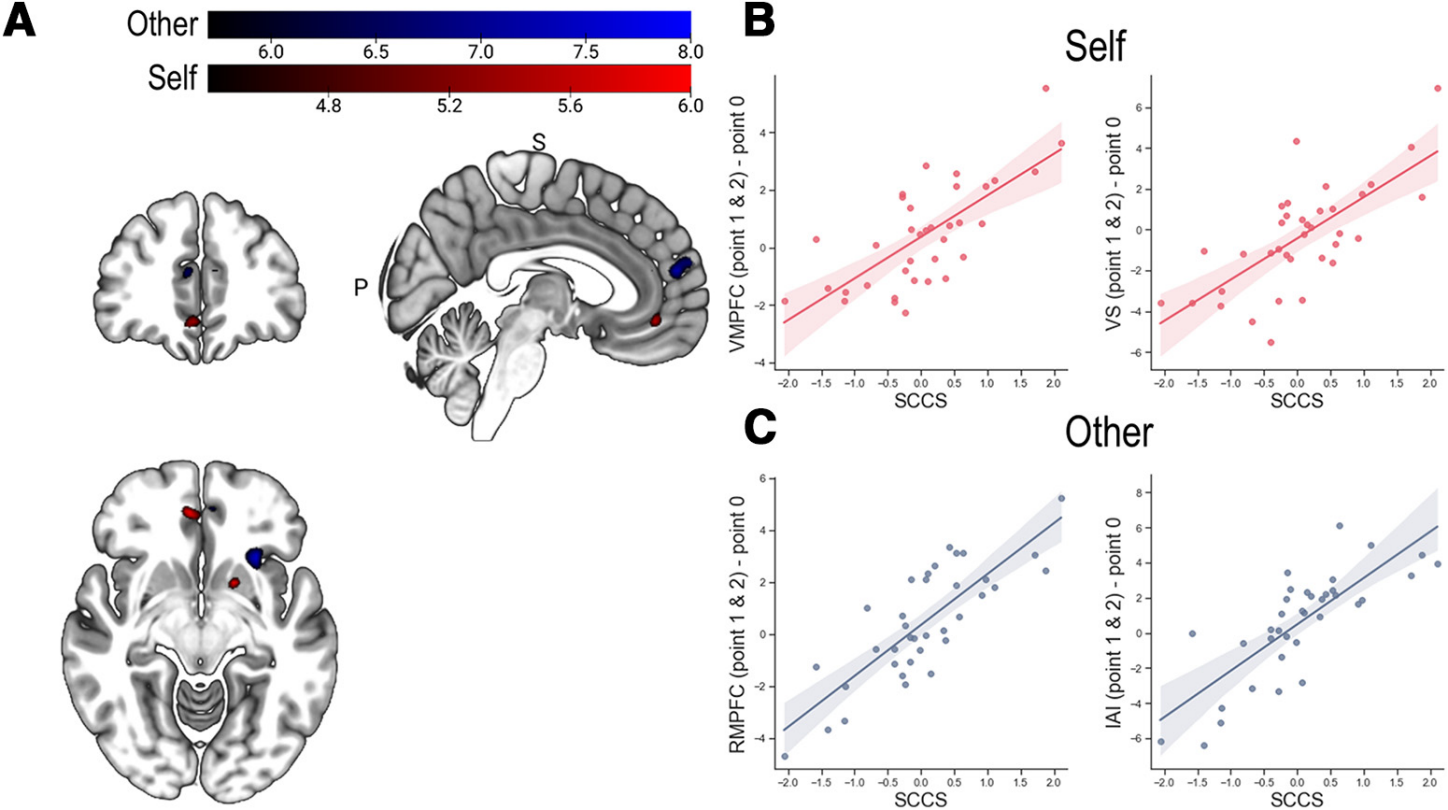
Correlation with SCCS for Self and Other conditions. ***A***, Whole-brain regression analyses where the contrast maps of [Point 1 and 2] versus Point 0 were regressed against the individuals' SCCS show the clusters with positive correlations for Self (red) and Other (green) conditions. ***B***, vmPFC and VS activities, showing positive correlations with the SCCS for the Self condition. ***C***, AI and rmPFC activities showed positive correlations with the SCCS for Other condition.

We also examined whether and how selfish motivation in Pareto lies is differentially associated with the neural representations in self- and other-benefiting dishonest opportunities. To achieve this, we regressed the SCCS on the contrast map of the Self and Other conditions separating them into two multiple regression analyses. During the Self condition, the activities in the vmPFC (*x* = 8, *y* = 44, *z* = −10) and the ventral striatum (VS: *x* = −16, *y* = 8, *z* = −8) showed significant positive correlations with the SCCS (Fig. 7*B*). This suggests that, as individuals consider opportunities for Both to be closer to opportunities for Self, self-benefiting dishonest opportunities engaged vmPFC and VS to a larger extent. During the Other condition, a significant positive correlation was observed between individual SCCS and the activities in vmPFC (*x* = −6, *y* = 48, *z* = −6) and VS (*x* = −18, *y* = 8, *z* = −2), similar to the observation made for the Self condition. Unlike the Self condition, however, the rmPFC (*x* = 6, *y* = 52, *z* = 16) and left anterior insula (AI: *x* = −26, *y* = 20, *z* = −12) additionally showed significant positive correlations with the SCCS during the Other condition (Fig. 7*C*). These findings indicate that other-benefiting dishonesty additionally engages rmPFC and AI among those with selfish motivation in Pareto lies.

## Discussion

This study proposed to infer individuals' covert primary motivations behind dishonesty based on neuroimaging data by adopting the brain-fingerprinting approach combined with machine-learning. As expected, the exhibited dishonest decisions that profit both the liar and others were identical regardless of the underlying motivation to benefit both. The individual measure of selfish motivation in Pareto white lies was estimated by the degree to which the multivoxel neural representation in the mPFC during the Both condition matches that during Self versus Other condition. The same measures showed positive correlations with the mean level of activity in the vmPFC and the rmPFC during the Both condition. Further RSAs demonstrated that higher selfish motivation in Pareto white lies can be characterized specifically by increased pattern matching between the Both and Self conditions in the vmPFC, and decreased pattern matching between the Both and Other conditions in the rmPFC. In addition, these neural findings were also mirrored by the behavioral data such that a higher degree of selfish motivation in Pareto lies, as measured by the increased pattern similarity between Self and Both condition in the vmPFC, was associated with faster RTs in Pareto versus altruistic lies, indicating qualitatively different processes subserving altruistic and Pareto lies. In summary, these findings suggest that hidden selfish motivation in white lies can be revealed by neural representation in the mPFC, and increased recruitment, as well as distinctive multivoxel neural patterns, of the vmPFC and the rmPFC characterize selfish motivation in Pareto lies.

Our *a priori* goal of this study was to identify the neural signatures of selfish motivation for Pareto lies. The higher the degree to which multivoxel neural representation in the mPFC during the Both condition matches that of Self than Other condition, the larger the mean activity observed in the vmPFC and VS when encountering opportunities for selfish gain. Moreover, the degree of pattern similarity in the vmPFC between Self and Both conditions predicted faster RTs for Pareto lies, the possible indicator of impulsive motivation for earning points by lying. Given the well-known functions of vmPFC in processing reward-predicting information (Knutson et al., 2000; O'Doherty et al., 2001; Kim et al., 2011) and intuitive valuation for decision-making (Shenhav and Greene, 2010; Tricomi et al., 2010; Buckholtz and Marois, 2012; Crockett, 2013; Janowski et al., 2013; Sul et al., 2015; Zaki and Cikara, 2015; Jung et al., 2018), these findings suggest that the increased mean activity in the vmPFC, as well as its specific multivoxel representational pattern that is shared between Self and Both conditions, is the core neural evidence and signatures of selfish motivation in Pareto white lies.

Unlike the vmPFC where the mean activity was positively correlated with the SCCS in all three conditions, the higher activity in the rmPFC and AI was positively associated with the SCCS when encountering opportunities for altruistic lies and Pareto white lies, but not for selfish lies. It was recently suggested that the mPFC can be hierarchically organized such that the rmPFC utilizes additional external sensory information from the environment to predict and prevent conflicts occurring in vmPFC tuned to internal bodily signals (Kim, 2020). Consistent with this idea, whereas vmPFC is involved in the internalized/intuitive social valuation, rmPFC contributes to the arbitration between internal and external valuation, playing a key role in context-dependent strategic valuation for social decision-making (Tusche et al., 2016; Jung et al., 2018; Yoon et al., 2018; Cutler and Campbell-Meiklejohn, 2019; Fukuda et al., 2019), including sophisticated and socially appropriate expression of self-protective behavior (Kumaran et al., 2016; Will et al., 2017; Yoon et al., 2018) and socially desirable behavior under social observation (Izuma et al., 2010; Jung et al., 2018; Yoon et al., 2021). Based on these theoretical and empirical studies, we can infer that those with higher selfish motivation in Pareto white lies can be characterized by increased intuitive/impulsive motivation subserved by the vmPFC and VS when considering dishonesty for Self condition, and also by an increased strategic regulation of such intuitive motivation subserved by the rmPFC and the AI when considering dishonesty for Other and Both.

**Figure 8.**
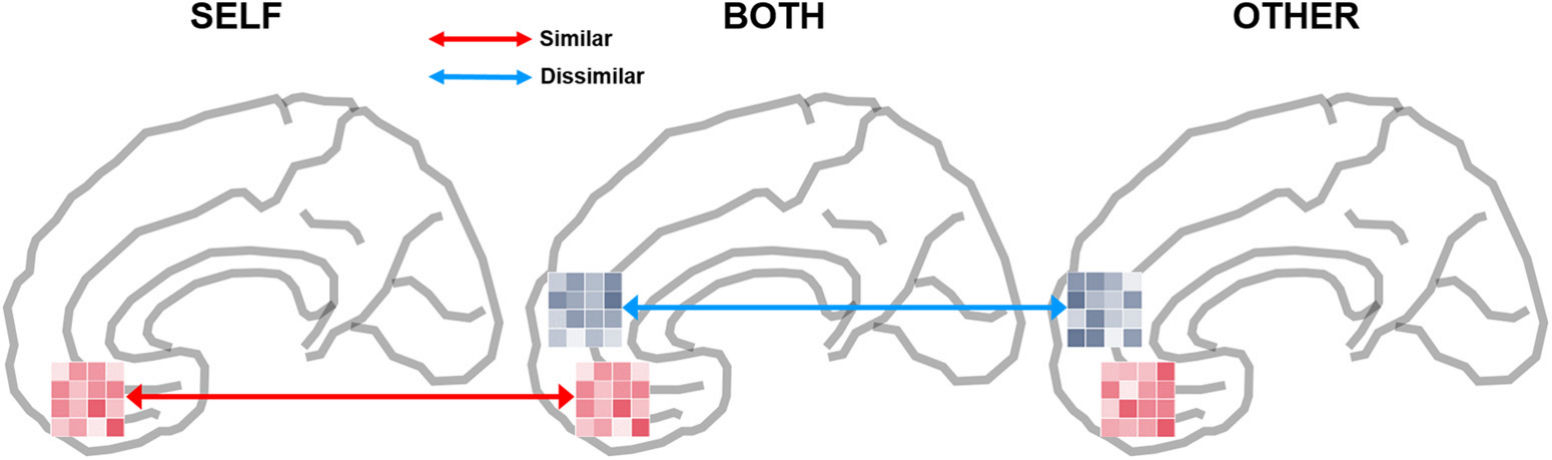
A schematic diagram of neural signatures in the mPFC associated with increased selfish motivation for Pareto lies. Individuals with higher selfish motivation in Pareto white lies are characterized by increased vmPFC activity when considering dishonesty in all three conditions and increased rmPFC activity when considering dishonesty for Other and Both. In addition, their neural representations in the vmPFC were similar between selfish and Pareto lying opportunities, but those in the rmPFC were dissimilar between altruistic and Pareto lying opportunities.

We also ran RSA on the two ROIs found in the univariate analysis and demonstrated that the representational similarity between Self and Both in the vmPFC and the representational dissimilarity between Other and Both in the rmPFC among those with higher SCCS indicating increased selfish motivation of Pareto white lies. Combining these findings with the univariate analysis results, we present the following two arguments. First, the rmPFC clusters showing increased mean activity in both Other and Both conditions may involve distinct neuronal ensembles, each serving different functions. Second, the distinct neuronal ensemble in rmPFC engaged in the Both condition, but not in Other condition, may increase the representational similarity between Self and Both in the vmPFC cluster (Fig. 8).

Those with higher degrees of Self-Both similarity in the vmPFC showed faster RT in the Both condition without observable difference in the proportion of dishonesty. This is consistent with the previous findings showing that neural activity related to dishonesty goes in parallel with RT, but not with dishonest behavior (Abe et al., 2018). Increased selfish motivation in Pareto lies likely minimizes conflicts, caused by multiple competing motivations when considering opportunities to lie for both oneself and others. However, those with a greater similarity between Self and Both conditions in the vmPFC activity pattern showed slower RT in being dishonest in Other versus Both condition, potential evidence for qualitatively different mental processes engaged for altruistic and Pareto lies among those with a higher degree of selfish motivation in Pareto lies.

In the univariate analyses, a more posterior cluster in the rmPFC, close to the pregenual ACC (Vogt, 2005), showed the highest activity when the beneficiary was Self and the lowest when the beneficiary was Both. The same region was also more active when more points were available. This activity may not be related to the increased motivation for dishonesty because participants lied more for Both than for Other even to the level of Self, which is opposite to the pattern of neural activity in this region across conditions. This observation led to a more plausible speculation that the activity in this region reflects a conflict between the urge to gain points and the guilt resulting from dishonesty, which is in line with the previous research showing increased ACC activity associated with moral conflict or guilt (Fourie et al., 2014; Abe et al., 2018). The fact that this region showed the lowest activity in the Both condition suggests that people experience the least moral conflict when dishonesty can benefit both the liar and another person. In addition, the activity in this region was also stronger among those with higher SCCS, possibly reflecting an increased moral conflict or guilt because of higher selfish motivation for Pareto lies.

We found no evidence for neural signatures of altruistic motivation for Pareto lies because there was no cluster in the brain showing a negative correlation with the SCCS even at a lenient threshold (*p* < 0.005 uncorrected). It has been established that the magnitude of the BOLD response is sensitive to change in excitation-inhibition balance in the cortical microcircuits involving the pyramidal projection neurons interacting with local GABAergic interneurons, which may reflect mismatch or prediction error-related feedback signals (Logothetis, 2008). Given this, larger negative SCCS, or higher Other-classification confidence score may not necessarily involve significant increase in excitation-inhibition balance because the multivoxel representation analysis can be immune to such a change in excitation-inhibition balance (Logothetis, 2008).

This study provides a novel methodological approach combining the potential benefits of univariate and multivariate analyses. Despite its superior sensitivity to detecting subtle differences in neural representation among different psychological states, multivariate pattern analysis has not been considered appropriate for identifying the exact neural mechanisms leading to the psychological state at question (Kohoutová et al., 2020), and insensitive to intersubject variability in mean activation across voxels within an ROI, which can be better captured by a conventional univariate analysis (Davis et al., 2014). Consistent with the dissociation between univariate and multivariate analyses, multivariate patterns showed a higher similarity of Both to Self versus Other, whereas univariate patterns showed the opposite, that is, the higher similarity of Both to Other versus Self, with the rmPFC clusters additionally recruited in Both and Other. This study demonstrated that a univariate analysis can be combined with multivariate pattern analysis to effectively locate the neural regions where the neural representations contributed maximally to the global pattern classification.

In conclusion, this study demonstrates that fMRI can be used to infer hidden selfish motivation in Pareto white lies by adopting the brain fingerprinting approach combining both univariate and multivariate analyses. This technique allowed us to estimate individual differences in motivation for Pareto lies, based on distinctive patterns of activity across functionally dissociable mPFC subregions, including vmPFC and rmPFC. We believe that this study will provide a novel and powerful research method and theoretical contributions to the current efforts of understanding complex motivations underlying moral behaviors.
